# The *c*-Differential-Linear Connectivity Table of Vectorial Boolean Functions

**DOI:** 10.3390/e26030188

**Published:** 2024-02-22

**Authors:** Said Eddahmani, Sihem Mesnager

**Affiliations:** 1 Department of Mathematics, University of Paris VIII, F-93526 Paris, France; said.eddahmani@etud.univ-paris8.fr; 2 Laboratory Geometry, Analysis and Applications (LAGA), University Sorbonne Paris Nord, CNRS, UMR 7539, F-93430 Villetaneuse, France; 3 Telecom Paris, Polytechnic Institute, F-91120 Palaiseau, France

**Keywords:** differential uniformity, vectorial function, S-box, linear codes, minimal codes

## Abstract

Vectorial Boolean functions and codes are closely related and interconnected. On the one hand, various requirements of binary linear codes are needed for their theoretical interests but, more importantly, for their practical applications (such as few-weight codes or minimal codes for secret sharing, locally recoverable codes for storage, etc.). On the other hand, various criteria and tables have been introduced to analyse the security of S-boxes that are related to vectorial Boolean functions, such as the Differential Distribution Table (DDT), the Boomerang Connectivity Table (BCT), and the Differential-Linear Connectivity Table (DLCT). In previous years, two new tables have been proposed for which the literature was pretty abundant: the *c*-DDT to extend the DDT and the *c*-BCT to extend the BCT. In the same vein, we propose extended concepts to study further the security of vectorial Boolean functions, especially the *c*-Walsh transform, the *c*-autocorrelation, and the *c*-differential-linear uniformity and its accompanying table, the *c*-Differential-Linear Connectivity Table (*c*-DLCT). We study the properties of these novel functions at their optimal level concerning these concepts and describe the *c*-DLCT of the crucial inverse vectorial (Boolean) function case. Finally, we draw new ideas for future research toward linear code designs.

## 1. Introduction

Vectorial Boolean functions are intensively used to produce S-boxes in block ciphers such as DES [1], Rinjdael or AES [2], Blowfish [3], GOST [4], and Serpent [5]. Various criteria have been proposed to test the resistance of S-boxes and the corresponding vectorial Boolean functions to known cryptanalytical attacks, such as the differential attack [6], the linear attack [7], and some of their variants.

Let $F:F_{2^{n}}\to F_{2^{m}}$ be a (n,m)-vectorial Boolean function. The derivative of *F* in the direction of $a\in F_{2^{n}}$ is the function $D_{a}\left(F\right)\left(x\right)=F\left(x\right)+F\left(x+a\right).$ The derivative is used to analyse the resistance of a vectorial Boolean function to the differential attack [6] and serves to build the Differential Distribution Table (DDT). The derivative is also used in the Boomerang Connectivity Table (BCT) [8] and in the Differential-Linear Connectivity Table (DLCT) [9,10]. The entry at $\left(a,b\right)\in F_{2^{n}}\times F_{2^{m}}$ of the DDT is defined by

$${DDT}_{F}\left(a,b\right)=\#\left\{x\in F_{2^{n}}:F\left(x\right)+F\left(x+a\right)=b\right\}.$$

To measure the resistance of a vectorial Boolean function, Nyberg [11] introduced the differential uniformity as

$$\delta_{F}=\max\left\{{DDT}_{F}\left(a,b\right)\;|\;\left(a,b\right)\in F_{2^{n}}\times F_{2^{m}},\;\mathrm{and}\;a\neq 0\right\}.$$

The most resistant vectorial Boolean functions have small differential uniformities. The reader can consult the [12] for a complete background on vectorial Boolean functions with a deep analysis of their cryptographic aspects.

At FSE 2002, Borisov et al. [13] proposed a variant of the differential attack to study ciphers’ resistance based on using modular multiplication as a primitive operation. This motivated Ellingsen et al. [14] to introduce the concept of *c*-differentials to study the resistance of a vectorial Boolean function to multiplicative variants of the differential attack. For a vectorial Boolean function $F:F_{2^{n}}\to F_{2^{m}}$ and $c\in F_{2^{m}}$, the *c*-derivative *F* with respect to $a\in F_{2^{n}}$ is the (n,m)-vectorial Boolean function DacF defined by DacF(x)=F(x+a)+cF(x) for all $x\in F_{2^{n}}$. The *c*-derivative is used to study the resistance of ciphers based on popular vectorial Boolean functions such as the inverse function [15], the Gold function [16], and various other functions [17,18,19,20,21]. As for the DDT, a *c*-differential table was proposed in [14], where the entry at $\left(a,b\right)\in F_{2^{n}}\times F_{2^{m}}$ is defined by

DDTFc(a,b)=#x∈F2n|F(x+a)+cF(x)=b.

Also, a *c*-differential uniformity was proposed in [14] by

δFc=maxDDTFc(a,b)|(a,b)∈F2n×F2m,anda≠0ifc=1.

The construction of functions, particularly permutations, with low *c*-differential uniformity is an interesting problem, and recent work has focused heavily on this direction. Likewise, regarding the original notion of differential uniformity leading to optimal functions Perfect Nonlinear (PN) and Almost Perfect Nonlinear (APN) over finite fields in odd and even characteristics, respectively, optimal functions having the lowest possible values of a *c*-differential uniformity have also been introduced. One can refer to [19,22,23,24,25,26,27] and the references therein. Some of those functions with low *c*-differential uniformity have been investigated. There are relatively few known (non-trivial, nonlinear) optimal classes of P*c*N and AP*c*N functions over finite fields with an even characteristic (see, e.g., [18,28,29,30,31] and the references therein).

Another popular cryptanalysis attack on S-boxes derived from Boolean functions is the boomerang attack, proposed by Wagner [32] in 1999. In connection with the boomerang attack, Cid et al. [8] proposed the Boomerang Connectivity Table (BCT) for a vectorial Boolean function where the entry at $\left(a,b\right)\in F_{2^{n}}\times F_{2^{m}}$ is defined by

$${BCT}_{F}\left(a,b\right)=\#\left\{x\in F_{2^{n}}:F^{-1}\left(F\left(x\right)+b\right)+F^{-1}\left(F\left(x+a\right)+b\right)=a\right\}.$$

Based on the BCT, Boura and Canteaut [33] introduced the boomerang uniformity of a vectorial Boolean function to measure its resistance against boomerang attack. The boomerang uniformity of *F* is defined by

$$\beta_{F}=\max_{a\in F_{2^{n}}^{*},b\in F_{2^{m}}^{*}}{BCT}_{F}\left(a,b\right).$$

To extend the BCT and the boomerang uniformity of a vectorial Boolean function, Stǎnicǎ [34] introduced the concept of the *c*-Boomerang Connectivity Table (*c*-BCT). For $c\in F_{2^{m}}^{*}$, the *c*-BCT is defined at the entry $\left(a,b\right)\in F_{2^{n}}\times F_{2^{m}}$ by

BCTFc(a,b)=#{x∈F2n:F−1(cF(x)+b)+F−1c−1F(x+a)+b=a}.

The corresponding *c*-boomerang uniformity is defined by

βFc=maxa∈F2n∗,b∈F2m∗BCTFc(a,b).

More generalizations of the differential and boomerang uniformities can be found in [35].

In 2019, Bar-On et al. [10] (see also [9]) introduced the Differential-Linear Connectivity Table (DLCT) of a vectorial Boolean function where the entry at $\left(a,b\right)\in F_{2^{n}}\times F_{2^{m}}$ is defined by

$${\mathrm{DLCT}}_{F}\left(a,b\right)=\#\left\{x\in F_{2^{n}}\;|\;b\cdot \left(F\left(x+a\right)+F\left(x\right)\right)=0\right\}-2^{n-1},$$

where x·y is the inner product of *x* and *y* on $F_{2^{m}}$. To measure the resistance of an S-box connected to a vectorial Boolean function, the differential-linear uniformity of *F* can be used, as defined by Li et al. in [36],

$$\gamma_{F}=\max_{a\in F_{2^{n}}^{*},b\in F_{2^{m}}^{*}}\left|{\mathrm{DLCT}}_{F}\left(a,b\right)\right|.$$

Various links exist between the DLCT and the Autocorrelation Table (ACT) of a vectorial Boolean function *F*. The ACT is defined at $\left(a,b\right)\in F_{2^{n}}\times F_{2^{m}}$ by

$${ACT}_{F}\left(a,b\right)=\sum_{x\in F_{2^{n}}}{\left(-1\right)}^{b\cdot (F(x)+F(x+a))}.$$

The corresponding absolute indicator is defined as

$$\Delta_{F}=\max_{\begin{array}{c} u\in F_{2^{n}},u\neq 0,\\ b\in F_{2^{m}}^{*} \end{array}}\left|{ACT}_{F}\left(a,b\right)\right|.$$

In [37], Canteaut et al. showed that the DLCT and the ACT of a vectorial Boolean function satisfy $\gamma_{F}=\frac{1}{2}\Delta_{F}$ and ${\mathrm{DLCT}}_{F}\left(a,b\right)=\frac{1}{2}{ACT}_{F}\left(a,b\right)$ for all $\left(a,b\right)\in F_{2^{n}}\times F_{2^{m}}$.

One can observe that the derivative $D_{a}\left(F\right)\left(x\right)=F\left(x\right)+F\left(x+a\right)$ of a Boolean function *F* is used in various tables, such as the DDT, the BCT, and the DLCT. Motivated by the crucial role of the derivative in the former tables and the attacks related to them, we propose three new concepts towards the *c*-derivative Dac(F)(x)=F(x+a)+cF(x):

• The *c*-Walsh transform of a vectorial Boolean function *F*: For $c\in F_{2^{m}}^{*}$, it is defined for $a\in F_{2^{n}}$ and $b\in F_{2^{m}}$ by
  WFc(a,b)=∑x∈F2n(−1)a·x+b·cF(x).
• The *c*-autocorrelation of a vectorial Boolean function: Let $c\in F_{2^{m}}$, c≠0. The *c*-autocorrelation of *F* at $\left(a,b\right)\in F_{2^{n}}\times F_{2^{m}}$ is the integer
  ACFc(a,b)=∑x∈F2n(−1)b·(F(x+a)+cF(x)).
  The absolute indicator is
  ΔFc=maxu∈F2n,u≠0ifc=1,b∈F2m∗ACFc(a,b),
  and the autocorrelation spectrum is
  ΛFc=ACFc(a,b),a∈F2n∗,b∈F2m∗.
• The *c*-Differential-Linear Connectivity Table (*c*-DLCT) where we use the *c*-derivative: Let $c\in F_{2^{m}}^{*}$. The *c*-DLCT of *F* is a $2^{n}\times 2^{m}$ table where the entry at $\left(a,b\right)\in F_{2^{n}}\times F_{2^{m}}$ is defined by
  DLCTFc(a,b)=#x∈F2n|b·(F(x+a)+cF(x))=0−2n−1.
  We also define the *c*-differential-linear uniformity of *F* as
  γFc=maxu∈F2n,u≠0ifc=1,b∈F2m∗DLCTFc(a,b),
  and, also, we define the *c*-DLCT spectrum of *F* by
  ΓFc=DLCTFc(a,b),a∈F2n,b∈F2m.

We show that there are numerous relationships between the three new concepts. Typically, we show that DLCTFc(a,b)=12ACFc(a,b) for all $\left(a,b\right)\in F_{2^{n}}\times F_{2^{m}}$ and γFc=12ΔFc.

Moreover, we focus on the inverse function defined on $F_{2^{n}}$ by $F\left(x\right)=\frac{1}{x}$ if x≠0, and F(0)=0. We study its *c*-DLCT and give an explicit value for the entries, including when c=1.

We mention that there is an interesting connection between *c* differential uniformity and combinatorial designs, which has been highlighted in [38] by showing that the graph of a perfect *c*-nonlinear function (an optimal function concerning the *c* differential uniformity) is a set of differences in a quasigroup. Difference sets give rise to symmetric designs, which are known to build optimal self-complementary codes. Some types of designs also have concrete applications such as secret sharing and visual cryptography.

Finally, we emphasise that one of our practical applications in brother research lines is to use the derived (optimal) functions (see, e.g., [12]) to derive minimal binary linear codes (see, e.g., [39]) that are needed for their theatrical interests but, more importantly, for their practical applicants such as few-weight codes or minimal codes for secret sharing and securing two-party computation.

The rest of this paper is organized as follows. Section 2 presents some known results that will be used in this paper. In Section 3, we define the *c*-Walsh and the *c*-autocorrelation of a vectorial Boolean function and study some of their properties. In Section 4, we present the concept of the *c*-DLCT and study its properties. We investigate the *c*-DLCT of the inverse function in Section 5. Finally, Section 6 concludes the paper and presents new ideas for future research toward linear code designs along the same lines as designing (minimal) codes from Almost Perfect Nonlinear (APN) and recent achievements [40] on minimal codes from low differential uniformity.

## 2. Preliminaries

In this section, we present some results and definitions that will be used in the next sections, including the *c*-derivative and the *c*-differential uniformity of a vectorial Boolean function.

For $b\in F_{2^{n}}$, we define the orthogonal space $b^{⊥}$ of *b* as follows.

**Definition** **1.** *For $b\in F_{2^{n}}$, the orthogonal space $b^{⊥}$ of b is defined by*

$$b^{⊥}=\left\{x\in F_{2^{n}}\;|\;b\cdot x=0\right\},$$

*where b·x is the inner product of b and x on $F_{2^{n}}$.*

The following result gives an explicit value for $\#b^{⊥}$.

**Proposition** **1.** *For $b\in F_{2^{n}}$, the orthogonal space $b^{⊥}$ of b satisfies*

$$\#b^{⊥}=\left\{\begin{array}{ll} 2^{n} & \mathrm{if}\;b=0,\\ 2^{n-1} & \mathrm{if}\;b\neq 0. \end{array}\right.$$

**Proof.**  It is obvious that $\#0^{⊥}=2^{n}$. Suppose that b≠0. Then, the binary expansion of *b* is in the following form.

$$b=(b_{n-1},b_{n-2},\ldots ,b_{j},\ldots ,b_{0}).$$

Suppose that $b_{j}=1$ for some *j* with 0≤j≤n−1. Let $x\in F_{2^{n}}$ such that $x\notin b^{⊥}$, that is b·x=1, with the binary expansion

$$x=(x_{n-1},x_{n-2},\ldots ,x_{j},\ldots ,x_{0}).$$

Let $y\in F_{2^{n}}$ with the binary expansion

$$y=(y_{n-1},y_{n-2},\ldots ,x_{j}+1\;\left(\mathrm{mod}\;2\right),\ldots ,x_{0}).$$

Then,

$$b\cdot y=b\cdot x+b_{j}\equiv 1+1\equiv 0\;\left(\mathrm{mod}\;2\right).$$

Hence, $y\in b^{⊥}$. It follows that for b≠0, each element *x* of $F_{2^{n}}$ satisfying b·x=1 is in correspondence with one element *y* of $F_{2^{n}}$ satisfying b·y=0. As a consequence, we have $\#b^{⊥}=2^{n-1}$. □

For n≥1, let $F_{2^{n}}$ be the finite field with $2^{n}$ elements. The trace of an element $x\in F_{2^{n}}$ is given by

$$\mathrm{Tr}\left(x\right)=x+x^{2}+\cdots +x^{2^{n-1}},$$

and satisfies Tr(x)∈{0,1}. The trace function satisfies $\mathrm{Tr}\left(x^{2}\right)=\mathrm{Tr}\left(x\right)$ for all $x\in F_{2^{n}}$.

The following lemma is well known and is useful for our work.

**Lemma** **1.** *Let n and k be positive integers and e=gcd(k,n). Then,*

$$\gcd\left(2^{k}+1,2^{n}-1\right)=\left\{\begin{array}{ll} 1 & \mathrm{if}\;\frac{n}{e}\;\mathrm{is}\;\mathrm{odd},\\ 2^{e}+1 & \mathrm{if}\;\frac{n}{e}\;\mathrm{is}\;\mathrm{even}. \end{array}\right.$$

Some specific equations on $F_{2^{n}}$ may be involved. The following result deals with the quadratic equation.

**Lemma** **2.** *(Proposition 1 of* [41]*) Let $a,b,c\in F_{2^{n}}$. The equation $ax^{2}+bx+c=0$ has*

(*i*) *One root if and only if b=0.*
(*ii*) *Two roots if and only if b≠0 and $\mathrm{Tr}\left(\frac{ac}{b^{2}}\right)=0$.*
(*iii*) *No root if and only if b≠0 and $\mathrm{Tr}\left(\frac{ac}{b^{2}}\right)=1$.*

The following lemma concerns another equation on $F_{2^{n}}$.

**Lemma** **3.** *Let k and n be positive integers such that k<n. Let d=gcd(k,n), $m=\frac{n}{d}>1$, and $\beta_{m-1}={\mathrm{Tr}}_{d}^{n}\left(B\right)$. Then, the trinomial $f\left(X\right)=X^{2^{k}}+X+B$ has no root if $\beta_{m-1}\neq 0$ and has $2^{d}$ roots x+δτ in $F_{2^{n}}$ if $\beta_{m-1}=0$, where $\delta \in F_{2^{d}}$, $\tau \in F_{2^{n}}$ is any element satisfying $\tau^{2^{k}-1}=1$, and*

$$x=\frac{1}{{\mathrm{Tr}}_{d}^{n}\left(c\right)}\sum_{i=0}^{m-1}\left(\sum_{j=0}^{i}c^{2^{kj}}\right)B^{2^{ki}},$$

*with any $c\in F_{2^{n}}^{*}$ satisfying ${\mathrm{Tr}}_{d}^{n}\left(c\right)\in F_{2^{d}}^{*}$.*

In [14], Ellingsen et al. proposed the concept of *c*-differentials. The following definitions are valid for binary finite fields.

**Definition** **2.** *Let $F:F_{2^{n}}\to F_{2^{m}}$ be an (n,m)-vectorial Boolean function and $c\in F_{2^{m}}$. The c-derivative F with respect to $a\in F_{2^{n}}$ is the (n,m)-vectorial function DacF satisfying pxt*

DacF(x)=F(x+a)+cF(x)

*for all $x\in F_{2^{n}}$.*

**Definition** **3.** *Let $F:F_{2^{n}}\to F_{2^{m}}$ be a (n,m)-vectorial Boolean function, and $c\in F_{2^{m}}$. The c-differential table of F is an $2^{n}\times 2^{m}$ table whose components are defined for $a\in F_{2^{n}}$ and $b\in F_{2^{m}}$ by*

ΔFc(a,b)=#{x∈F2n|F(x+a)+cF(x)=b}.

**Definition** **4.** *Let $F:F_{2^{n}}\to F_{2^{m}}$ be a (n,m)-vectorial Boolean function, and $c\in F_{2^{m}}$. The c-differential uniformity of F is*

ΔFc=maxa∈F2n,b∈F2mΔFc(a,b)ifc≠1,maxa∈F2n∖{0},b∈F2mΔFc(a,b)ifc=1.

## 3. The c-Walsh and c-Autocorrelation of a Vectorial Boolean Function

The Walsh transform of a Boolean function $f:F_{2^{n}}\to F_{2}$ is defined at $u\in F_{2^{n}}$ by

$$W_{f}\left(u\right)=\sum_{x\in F_{2^{n}}}{\left(-1\right)}^{u\cdot x+f(x)},$$

where u·x is the inner product of *u* and *x*. The Walsh transform serves to compute the linearity of *f* as

$$L\left(f\right)=\max_{u\in F_{2^{n}}}\left|W_{f}\left(u\right)\right|.$$

For a vectorial Boolean function $F:F_{2^{n}}\to F_{2^{m}}$, the Walsh transform of *F* is defined for $u\in F_{2^{n}}$ and $v\in F_{2^{m}}$ by

$$W_{F}\left(u,v\right)=\sum_{x\in F_{2^{n}}}{\left(-1\right)}^{u\cdot x+v\cdot F(x)},$$

and is used to compute the linearity of *F* by

$$L\left(F\right)=\max_{u\in F_{2^{n}},v\in F_{2^{n}}\setminus \left\{0\right\}}\left|W_{F}\left(u,v\right)\right|.$$

We extend the Walsh transform of a vectorial Boolean function to the *c*-Walsh transform as follows.

**Definition** **5.** *Let F be an (n,m)-vectorial Boolean function, and $c\in F_{2^{m}}^{*}$. The c-Walsh transform of F is defined for $u\in F_{2^{n}}$ and $v\in F_{2^{m}}$ by*

WFc(u,v)=∑x∈F2n(−1)u·x+v·cF(x).

The autocorrelation function is used to study various properties of the Boolean functions (see [42]).

**Definition** **6.** *Let f be Boolean function defined on $F_{2^{n}}$. The autocorrelation of f at $u\in F_{2^{n}}$ is the integer*

$${\mathrm{AC}}_{f}\left(u\right)=\sum_{x\in F_{2^{n}}}{\left(-1\right)}^{f(x)+f(x+u)},$$

*and its absolute indicator is $\Delta_{f}=\max_{u\in F_{2^{n}},u\neq 0}\left|{\mathrm{AC}}_{f}\left(u\right)\right|$.*

We notice that u=0 is excluded in the definition of the absolute indicator since ${\mathrm{AC}}_{f}\left(0\right)=\sum_{x\in F_{2^{n}}}{\left(-1\right)}^{f(x)+f(x)}=2^{n}$. The generalization of the autocorrelation to vectorial Boolean functions can be then defined as follows.

**Definition** **7.** *Let F be an (n,m)-vectorial Boolean function defined on $F_{2^{n}}$. The autocorrelation of F at $\left(u,v\right)\in F_{2^{n}}\times F_{2^{m}}$ is the integer*

$${\mathrm{AC}}_{F}\left(u,v\right)=\sum_{x\in F_{2^{n}}}{\left(-1\right)}^{v\cdot (F(x)+F(x+u))}.\;$$

*The absolute indicator is*

$$\Delta_{F}=\max_{\begin{array}{c} u\in F_{2^{n}},u\neq 0,\\ v\in F_{2^{m}},v\neq 0 \end{array}}\left|{\mathrm{AC}}_{F}\left(u,v\right)\right|,$$

*and the autocorrelation spectrum is*

$$\Lambda_{F}=\left\{{\mathrm{AC}}_{F}\left(u,v\right),u\in F_{2^{n}},u\neq 0,v\in F_{2^{m}},v\neq 0\right\}.$$

The trivial values are not considered in the definition of the absolute indicator since ${\mathrm{AC}}_{F}\left(0,v\right)={\mathrm{AC}}_{F}\left(u,0\right)=2^{n}$.

Inspired by Definition 6, we introduce the notion of *c*-autocorrelation of a Boolean function.

**Definition** **8.** *Let f be the Boolean function defined on $F_{2^{n}}$, and $c\in F_{2^{m}}$, c≠0. The c-autocorrelation of f at $u\in F_{2^{n}}$ is the integer*

ACfc(u)=∑x∈F2n(−1)f(x+u)+cf(x),

*and the c-absolute indicator is Δfc=maxu∈F2nACf(u).*

Similarly, to generalize Definition 7, we define the *c*-autocorrelation of a vectorial Boolean function.

**Definition** **9.** *Let F be an (n,m)-vectorial Boolean function defined on $F_{2^{n}}$, and $c\in F_{2^{m}}$, c≠0. The c-autocorrelation of F at $\left(u,v\right)\in F_{2^{n}}\times F_{2^{m}}$ is the integer*

ACFc(u,v)=∑x∈F2n(−1)v·(F(x+u)+cF(x)).

*The absolute indicator is*

ΔFc=maxu∈F2n,u≠0ifc=1,v∈F2m,v≠0ACFc(u,v),

*and the autocorrelation spectrum is*

ΛFc=ACFc(u,v),u∈F2n,v∈F2m,.

To ease the study of the *c*-autocorrelation of a vectorial Boolean function *F*, we present its *c*-autocorrelation table defined at $\left(u,v\right)\in F_{2^{n}}\times F_{2^{m}}$ by

ACTFc(u,v)=∑x∈F2n(−1)v·(F(x+u)+cF(x)).

The following result links the *c*-autocorrelation of a vectorial Boolean function and its *c*-Walsh transform.

**Proposition** **2.** *Let F be an (n,m) Boolean function. Then, for any $u\in F_{2^{n}}$ and any $v\in F_{2^{n}}$,*

WF(u,v)WFc(u,v)=∑z∈F2n(−1)u·zACFc(z,v).

**Proof.**  We have

WF(u,v)WFc(u,v)=∑x∈F2n(−1)u·x+v·F(x)∑y∈F2n(−1)u·y+v·cF(y)=∑x,y∈F2n(−1)u·(x+y)+v·(F(x)+cF(y)=∑y,z∈F2n(−1)u·z+v·(F(y+z)+cF(y))=∑z∈F2n(−1)u·z∑y∈F2n(−1)v·(F(y+z)+cF(y))=∑z∈F2n(−1)u·zACFc(z,v).

This finishes the proof. □

## 4. The c-Differential-Linear Connectivity Table of a Vectorial Boolean Function

In this section, we present a new concept, called the *c*-Differential-Linear Connectivity Table (*c*-DLCT), which generalizes the standard DLCT, independently defined in 2018 by Kim et al. [9] and Bar-On et al. [10].

We start by defining the standard Differential-Linear Connectivity Table (DLCT).

**Definition** **10.** *Let F be an (n,m)-vectorial Boolean function. The DLCT of F is an $2^{n}\times 2^{m}$ table where the entry at $\left(u,v\right)\in F_{2^{n}}\times F_{2^{m}}$ is*

$${\mathrm{DLCT}}_{F}\left(u,v\right)=\#\left\{x\in F_{2^{n}}\;|\;v\cdot \left(F\left(x+u\right)+F\left(x\right)\right)=0\right\}-2^{n-1}.$$

The DLCT is a tool that could analyse the relationships between differential and linear parts of a block cipher. One can observe that if $x\in F_{2^{n}}$ is such that v·(F(x+u)+F(x))=0, then v·(F((x+u)+u)+F(x+u))=0. Consequently, ${\mathrm{DLCT}}_{F}\left(u,v\right)$ is always even. Moreover, if u=0, or if v=0, then ${\mathrm{DLCT}}_{F}\left(u,v\right)=2^{n-1}$. This induces the following definition for differential-linear connectivity uniformity.

**Definition** **11.** *Let F be an (n,m)-vectorial Boolean function. The differential-linear connectivity uniformity of F is*

$$\gamma_{F}=\max_{u\in F_{2^{n}}^{*},v\in F_{2^{m}}^{*}}\left|{\mathrm{DLCT}}_{F}\left(u,v\right)\right|.$$

The DLCT of a vectorial Boolean function is related to the autocorrelation function by the following relation.

$$\begin{array}{cc} {AC}_{F}\left(u,v\right) & =\#\{x\in F_{2^{n}}\;|\;v\cdot \left(F\left(x\right)+F\left(x+u\right)\right)=0)\}\\ & \;-\#\{x\in F_{2^{n}}\;|\;v\cdot \left(F\left(x\right)+F\left(x+u\right)\right)=1\}\\ & =2\#\left\{x\in F_{2^{n}}\;|\;v\cdot \left(F\left(x\right)+F\left(x+u\right)\right)=0\right\}-2^{n}\\ & =2{\mathrm{DLCT}}_{F}\left(u,v\right). \end{array}$$

The DLCT is a tool to study the relationships between the linear and the differential properties of a block cipher. For $\left(u,v\right)\in F_{2^{n}}\times F_{2^{m}}$, it counts the number of elements $x\in F_{2^{n}}$ such that v·(F(x+u)+F(x))=0. Let $a\in F_{2^{m}}$, a≠0, and $b\in F_{2^{m}}$, b≠0, be two fixed non-zero elements. It is possible to study the relationships between the linear and the differential properties of a block cipher by studying the number of solutions of the equation v·(aF(x+u)+bF(x))=0 or equivalently v·(F(x+u)+cF(x))=0, where $c=\frac{a}{b}$. This leads us to define a function’s *c*-Differential-Linear Connectivity Table (*c*-DLCT).

**Definition** **12.** *Let F be an (n,m)-vectorial Boolean function, and $c\in F_{2^{m}}$, c≠0. The c-DLCT of F is an $2^{n}\times 2^{m}$ table where the entry at $\left(u,v\right)\in F_{2^{n}}\times F_{2^{m}}$ is*

DLCTFc(u,v)=#x∈F2n|v·(F(x+u)+cF(x))=0−2n−1.

*Moreover, the c-differential-linear connectivity uniformity of F is*

γFc=maxu∈F2n,u≠0ifc=1,v∈F2m,v≠0DLCTFc(u,v),

*and the c-DLCT spectrum of F is defined for $\left(u,v\right)\in F_{2^{n}}\times F_{2^{m}}$ by*

ΓFc=DLCTFc(u,v),u∈F2n,v∈F2m.

From Definitions 9 and 12, we obtain the following connection between the ACTc and the DLCTc of a vectorial Boolean function.

**Proposition** **3.** *Let F be (n,m)-vectorial Boolean function. Then, for all $u\in F_{2^{n}}$ and $v\in F_{2^{m}}$,*

DLCTFc(u,v)=12ACFc(u,v),andγFc=12ΔFc.

**Proof.**  We have

ACFc(u,v)=#{x∈F2n|v·(F(x+u)+cF(x))=0)}−#{x∈F2n|v·(F(x+u)+cF(x))=1}=2#{x∈F2n|v·(F(x+u)+cF(x))=0}−2n=2DLCTFc(u,v).

which gives DLCTFc(u,v)=12ACFc(u,v). On the other hand, we have

ΔFc=maxu∈F2n,u≠0ifc=1,v∈F2m,v≠0ACFc(u,v)=2maxu∈F2n,u≠0ifc=1,v∈F2m,v≠0DLCTFc(u,v)=2γFc,

and γFc=12ΔFc. This finishes the proof. □

As a consequence of the former proposition, the following result connects the *c*-DLCT and the *c*-derivative of a vectorial Boolean function via the Walsh transform.

**Proposition** **4.** *Let F be an (n,m)-vectorial Boolean function, and $c\in F_{2^{m}}$, c≠0. Then, for any $\left(u,v\right)\in F_{2^{n}}\times F_{2^{m}}$,*

DLCTFc(u,v)=12W(DucF)(0,v).

**Proof.**  Combining Definition 2 and the definition of the Walsh transform, we obtain

W(DucF)(0,v)=∑x∈F2n(−1)v·(F(x+u)+cF(x))=ACFc(u,v).

Then, using Proposition 3, we have

WDucF(0,v)=ACFc(u,v)=2DLCTFc(u,v),

and DLCTFc(u,v)=12W(DucF)(0,v). □

The following result shows a connection between the *c*-DLCT and the *c*-derivative of a vectorial Boolean function via the Walsh transform.

**Proposition** **5.** *Let F be an (n,m)-vectorial Boolean function, and $c\in F_{2^{m}}$, c≠0. Then, for any $\left(u,v\right)\in F_{2^{n}}\times F_{2^{m}}$,*

WF(u,v)WFc(u,v)=2∑ω∈F2n(−1)u·ωDLCTFc(ω,v).

**Proof.**  Combining Proposition 2 and Proposition 4, we obtain

WF(u,v)WFc(u,v)=∑z∈F2n(−1)u·zACFc(z,v)=2∑ω∈F2n(−1)u·ωDLCTFc(ω,v),

as claimed. □

The following result gives a link between DLCTFc and ΔFc(a,b).

**Proposition** **6.** *Let F be an (n,m)-vectorial Boolean function, and $c\in F_{2^{m}}$, c≠0. Then, for any $\left(u,v\right)\in F_{2^{n}}\times F_{2^{m}}$,*

DLCTFc(u,v)=12∑ω∈F2n(−1)ω·vΔFc(u,ω).

**Proof.**  By Proposition 3, we have

2DLCTFc(u,v)=ACFc(u,v)=#{x∈F2n|v·(F(x+u)+cF(x))=0)}−#{x∈F2n|v·(F(x+u)+cF(x))=1}=∑ω∈F2n,ω·v=0#{x∈F2n|F(x+u)+cF(x)=ω}−∑ω∈F2n,ω·v=1#{x∈F2n|F(x+u)+cF(x)=ω}=∑ω∈F2n(−1)ω·v#{x∈F2n|F(x+u)+cF(x)=ω}=∑ω∈F2n(−1)ω·vΔFc(u,ω).

This leads to

DLCTFc(u,v)=12∑ω∈F2n(−1)ω·vΔFc(u,ω),

which finishes the proof. □

## 5. The c-DLCT of the Inverse Function

In this section, we give the explicit values of the entries of the *c*-DLCT, including the case c=1, and give some numerical results on $F_{2^{n}}$ with 3≤n≤8.

### 5.1. The 1-DLCT of the Inverse Function

For c=1, the 1-DLCT satisfies the following result.

**Theorem** **1.** *Let $F:F_{2^{n}}\to F_{2^{n}}$ be the inverse function defined by F(0)=0, and $F\left(x\right)=x^{2^{n-2}}$ for x≠0. For $a,b\in F_{2^{n}}$, define the set*

$$\begin{array}{c} \begin{array}{cc} E_{0}\left(a,b\right) & =\left\{z\in b^{⊥}\;|\;z\neq 0,\mathrm{Tr}\left(\frac{1}{az}\right)=0\right\}, \end{array} \end{array}$$

*where $b^{⊥}$ is the orthogonal space of b. Then,*

DLCTF1(a,b)=2n−1ifa=0,orb=0,2#E0(a,b)+2−2n−1if1a∈b⊥,2#E0(a,b)−2n−1if1a∉b⊥.

**Proof.**  We use the definition

DLCTF1(a,b)=#x∈F2n|b·(F(x+a)+F(x))=0−2n−1.

We consider the following cases.

**Case** **1.**  Suppose that b=0. Then, for all $x\in F_{2^{n}}$, b·(F(x+a)+F(x))=0. Hence,

DLCTF1(a,0)=2n−2n−1=2n−1.

**Case** **2.**  Suppose that b≠0 and a=0. Then, for all $x\in F_{2^{n}}$, b·(F(x+a)+F(x))=b·0=0. This leads to

DLCTF1(0,b)=2n−2n−1=2n−1.

**Case** **3.**  Suppose that b≠0 and a≠0. Consider the equation

(1) b·(F(x+a)+F(x))=0.

**Case** **3.1.**  If x=0, then

$$b\cdot \left(F\left(x+a\right)+F\left(x\right)\right)=b\cdot F\left(a\right)=b\cdot \frac{1}{a}.$$

Hence, x=0 is a solution of the Equation (1) if and only if $\frac{1}{a}\in b^{⊥}$.

**Case** **3.2.**  If x=a, then

$$b\cdot \left(F\left(x+a\right)+F\left(x\right)\right)=b\cdot F\left(a\right)=b\cdot \frac{1}{a}.$$

Hence, x=a is a solution of the Equation (1) if and only if $\frac{1}{a}\in b^{⊥}$.

**Case** **3.3.**  Suppose that x≠0 and x≠a. We have

$$F\left(a+x\right)+F\left(x\right)=\frac{1}{a+x}+\frac{1}{x}=\frac{a}{x^{2}+ax}.$$

If b·(F(a+x)+F(x))=0, then F(a+x)+F(x)=z for some $z\in b^{⊥}$, that is $\frac{a}{x^{2}+ax}=z,$ or equivalently

(2) $$\begin{array}{c} zx^{2}+azx+a=0. \end{array}$$

**Case** **3.3.1.**  If z=0, then the Equation (2) reduces to a=0, which is not possible.

**Case** **3.3.2.**  Suppose that z≠0. If $\mathrm{Tr}\left(\frac{1}{az}\right)=1$, then, by Lemma 2, the Equation (2) has no solution, and if $\mathrm{Tr}\left(\frac{1}{az}\right)=0$, it has two solutions.

Define the set

$$\begin{array}{c} \begin{array}{cc} E_{0}\left(a,b\right) & =\left\{z\in b^{⊥}\;|\;z\neq 0,\mathrm{Tr}\left(\frac{1}{az}\right)=0\right\}. \end{array} \end{array}$$

The DLCT1 in Case 3 is then

DLCTF1(a,b)=2#E0(a,b)+2−2n−1if1a∈b⊥,2#E0(a,b)−2n−1if1a∉b⊥,

which finishes the proof. □

### 5.2. The *c*-DLCT of the Inverse Function for c≠1

**Theorem** **2.** *Let $F:F_{2^{n}}\to F_{2^{n}}$ be the inverse function defined by F(0)=0, and $F\left(x\right)=\frac{1}{x}$ for x≠0. Let $c\in F_{2^{n}}$ with c≠0 and c≠1. For $a,b\in F_{2^{n}}$, define the set*

$$\begin{array}{c} \begin{array}{cc} E_{0}\left(a,b,c\right) & =\left\{z\in b^{⊥}\;|\;z\neq 0,z\neq \frac{1+c}{a},\mathrm{Tr}\left(\frac{acz}{{\left(1+c+az\right)}^{2}}\right)=0\right\}, \end{array} \end{array}$$

*where $b^{⊥}$ is the orthogonal space of b. Then,*

DLCTFc(a,b)=2n−1ifb=0,0ifa=0,b≠0,2#E0(a,b,c)+2−2n−1if1a∈b⊥,ca∉b⊥,2#E0(a,b,c)+2−2n−1if1a∉b⊥,ca∈b⊥,2#E0(a,b,c)+4−2n−1if1a∈b⊥,ca∈b⊥,2#E0(a,b,c)+2−2n−1if1a∉b⊥,ca∉b⊥.

**Proof.**  Suppose that c≠0 and c≠1. We use the definition

DLCTFc(a,b)=#x∈F2n|b·(F(x+a)+cF(x))=0−2n−1.

We consider the following cases.

**Case 1.** Suppose that b=0. Then, for all $x\in F_{2^{n}}$, b·(F(x+a)+cF(x))=0. Hence,

DLCTFc(a,0)=2n−2n−1=2n−1.

**Case 2.** Suppose that b≠0 and a=0. If b·(F(x+a)+cF(x))=0, then b·(1+c)F(x)=0, and $\left(1+c\right)F\left(x\right)\in b^{⊥}$. Observe that x=0 is a possible solution. If x≠0, then there exists $z\in b^{⊥}\setminus \left\{0\right\}$ such that (1+c)F(x)=z, that is $\frac{1+c}{x}=z$, and $x=\frac{1+c}{z}$. This leads to

DLCTFc(0,b)=#b⊥−2n−1=0.

**Case** **3.**  Suppose that a≠0 and b≠0. Consider the equation

(3) b·(F(x+a)+cF(x))=0.

**Case** **3.1.**  If x=0, then

$$b\cdot \left(F\left(x+a\right)+cF\left(x\right)\right)=b\cdot F\left(a\right)=b\cdot \frac{1}{a}.$$

Hence, x=0 is a solution of the Equation (3) if and only if $\frac{1}{a}\in b^{⊥}$.

**Case** **3.2.**  If x=a, then

$$b\cdot \left(F\left(x+a\right)+cF\left(x\right)\right)=b\cdot cF\left(a\right)=b\cdot \frac{c}{a}.$$

Hence, x=a is a solution of the Equation (3) if and only if $\frac{c}{a}\in b^{⊥}$.

**Case** **3.3.**  Suppose that x≠0 and x≠a. We have

$$F\left(a+x\right)+cF\left(x\right)=\frac{1}{a+x}+\frac{c}{x}=\frac{(1+c)x+ac}{x^{2}+ax}.$$

If b·(F(a+x)+cF(x))=0, then F(a+x)+cF(x)=z for some $z\in b^{⊥}$, that is $\frac{(1+c)x+ac}{x^{2}+ax}=z,$ or equivalently

(4) $$\begin{array}{c} zx^{2}+\left(1+c+az\right)x+ac=0. \end{array}$$

**Case** **3.3.1.**  If z=0, then the Equation (4) reduces to (1+c)x+ac=0, which has one solution $x=\frac{ac}{1+c}$.

**Case** **3.3.2.**  If $z_{0}=\frac{1+c}{a}\in b^{⊥}$, then for $z_{0}$, the Equation (4) reduces to $z_{0}x^{2}+ac=0$, which, by Lemma 2, has one solution.

**Case** **3.3.3.**  Suppose that z≠0 and $z\neq \frac{1+c}{a}$. If $\mathrm{Tr}\left(\frac{acz}{{\left(1+c+az\right)}^{2}}\right)=1$, then, by Lemma 2, the Equation (4) has no solution, and if $\mathrm{Tr}\left(\frac{acz}{{\left(1+c+az\right)}^{2}}\right)=0$, it has two solutions.

To summarize all the cases, we define the set

$$\begin{array}{c} \begin{array}{cc} E_{0}\left(a,b,c\right) & =\left\{z\in b^{⊥}\;|\;z\neq 0,z\neq \frac{1+c}{a},\mathrm{Tr}\left(\frac{acz}{{\left(1+c+az\right)}^{2}}\right)=0\right\}. \end{array} \end{array}$$

The DLCTc in Case 3 is then

DLCTFc(a,b)=2#E0(a,b,c)+2−2n−1if1a∈b⊥,ca∉b⊥,2#E0(a,b,c)+2−2n−1if1a∉b⊥,ca∈b⊥,2#E0(a,b,c)+4−2n−1if1a∈b⊥,ca∈b⊥,2#E0(a,b,c)+2−2n−1if1a∉b⊥,ca∉b⊥,

which finishes the proof. □

### 5.3. Numerical Results for the c-DLCT of the Inverse Function

We have computed the *c*-DLCT of the inverse function over $F_{2^{n}}$ for 3≤n≤7, and all $c\in F_{2^{n}}^{*}$, while for n=8, we only compute it for c=1,2,…,10. The inversion and multiplication in $F_{2^{n}}$ are processed modulo the polynomials presented in Table 1.

In Table 2, we present the values of DLCTFc(u,v) of the inverse function over $F_{2^{4}}$ with c=0×9.

For the inverse function over $F_{2^{n}}$, we present in Table 3 the *c*-DLCT spectrum ΓFc and *c*-differential-linear uniformity γFc for 3≤n≤8 and for small values of *c*. All the other *c*-DLCT spectrums reduce to one of the listed ones in the table.

## 6. Conclusions

In this paper, we introduced and studied new cryptographic tools and parameters to help us quantify the security of S-boxes (mathematically, vectorial Boolean functions) involving block ciphers as main components: the *c*-Walsh transform, the *c*-autocorrelation, and the *c*-differential-linear uniformity. We also introduced a new table called the *c*-Differential-Linear Connectivity Table (*c*-DLCT) to analyse attacks related to the differential and the linear attacks. We considered various S-box family properties associated with the above-mentioned notion and presented the values of the *c*-DLCT of the particular crucial case of the inverse function. Finally, recall that codes over finite fields have been studied extensively because of their linear structures and practical implementations. It is the basis of the research on various kinds of codes. One well-known construction method of linear codes is derived from special functions (essentially from cryptographic functions which play a crucial role in symmetric cryptography) over finite fields (see the book [12]). Cryptographic multi-output Boolean functions and codes have essential data communication and storage applications. These two areas are closely related and have had a fascinating interplay (see, e.g., the book chapter in [43] and the references therein). Cryptographic functions and linear codes are closely related and have had a fascinating interplay. Cryptographic functions (e.g., highly nonlinear functions, Perfect Nonlinear (PN), Almost Perfect Nonlinear (APN), Bent, Almost Bent (AB), and Plateaued) have essential applications in coding theory. For instance, Perfect Nonlinear (APN or PN) functions have been employed to construct optimal linear codes (see, e.g., [44,45,46,47,48] and the references therein). Very recently, Mesnager, Shi, and Zhu [40] proposed several constructions of minimal (cyclic) codes from low differential uniform functions. Given these works, the derived functions from this paper would help design new families of binary minimal codes. We will keep an in-depth study of them in future work and cordially invite interested readers to investigate them.

## Figures and Tables

**Table 1:** The polynomials of $F_{2^{n}}$ for 3≤n≤8.

$F_{2^{n}}$	Polynomial
$F_{2^{3}}$	$x^{3}+x+1$
$F_{2^{4}}$	$x^{4}+x+1$
$F_{2^{5}}$	$x^{5}+x^{3}+1$
$F_{2^{6}}$	$x^{6}+x^{3}+1$
$F_{2^{7}}$	$x^{7}+x^{3}+1$
$F_{2^{8}}$	$x^{8}+x^{4}+x^{3}+x^{2}+1$

**Table 2:** The values of DLCTFc(u,v) of the *c*-DLCT of the inverse function over $F_{2^{4}}$ for c=0×9.

u∖v	0	1	2	3	4	5	6	7	8	9	a	b	c	d	e	f
0	8	0	0	0	0	0	0	0	0	0	0	0	0	0	0	0
1	8	0	2	2	0	−4	2	−2	2	−4	0	2	2	0	0	−2
2	8	2	0	−2	2	2	2	−2	0	2	−4	2	−4	0	0	0
3	8	0	2	0	−2	−4	0	0	−2	0	2	2	2	2	2	−4
4	8	−2	2	−2	0	2	−4	0	2	0	2	2	2	−4	0	0
5	8	2	0	2	0	0	−2	2	−4	0	2	2	−2	0	2	−4
6	8	0	−2	0	−2	2	2	−4	0	2	−4	0	0	2	2	2
7	8	2	−4	−4	2	−2	0	2	2	0	2	−2	0	0	2	0
8	8	−2	0	0	2	2	2	0	−2	2	2	−4	0	2	−4	0
9	8	2	−4	0	2	0	2	2	0	2	0	−4	2	0	−2	−2
a	8	2	0	2	−4	2	−2	−4	2	0	0	−2	0	2	0	2
b	8	−4	0	2	0	2	2	2	0	−2	0	0	−2	2	−4	2
c	8	0	−2	−4	0	0	0	2	0	−2	2	2	2	−4	2	2
d	8	0	2	2	−4	0	−4	0	2	2	0	0	2	−2	−2	2
e	8	−4	2	2	2	−2	0	0	2	−4	−2	0	0	2	0	2
f	8	2	2	0	2	0	0	2	−4	2	−2	0	−4	−2	2	0

**Table 3:** The *c*-DLCT spectrum and the *c*-differential-linear connectivity uniformity of the inverse function over $F_{2^{n}}$ for 3≤n≤8 and small *c*.

$F_{2^{n}}$	*c*	ΓFc	γFc
$F_{2^{3}}$	1	{−4,0,4}	4
$F_{2^{3}}$	2	{−2,0,2,4}	2
$F_{2^{4}}$	1	{−4,0,4,8}	4
$F_{2^{4}}$	2	{−4,−2,0,2,8}	4
$F_{2^{4}}$	6	{−2,0,2,4,8}	4
$F_{2^{5}}$	1	{−4,0,4,16}	4
$F_{2^{5}}$	2	{−6,−4,−2,0,2,4,6,16}	6
$F_{2^{5}}$	3	{−6,−4,−2,0,2,4,16}	6
$F_{2^{5}}$	7	{−4,−2,0,2,4,16}	4
$F_{2^{6}}$	1	{−8,−4,0,4,8,32}	8
$F_{2^{6}}$	2	{−8,−6,−4,−2,0,2,4,6,8,32}	8
$F_{2^{6}}$	6	{−8,−6,−4,−2,0,2,4,6,32}	8
$F_{2^{6}}$	8	{−6,−4,−2,0,2,6,8,32}	8
$F_{2^{7}}$	1	{−12,−8,−4,0,4,8,64}	12
$F_{2^{7}}$	2	{−12,−10,−8,−6,−4,−2,0,2,4,6,8,10,64}	12
$F_{2^{8}}$	1	{−16,−12,−8,−4,0,4,8,12,16,128}	16
$F_{2^{8}}$	2	{−16,−14,−12,−10,−8,−6,−4,−2,0,2,4,6,8,10,12,14,16,128}	16
$F_{2^{8}}$	6	{−16,−14,−12,−10,−8,−6,−4,−2,0,2,4,6,8,10,12,14,128}	16
$F_{2^{8}}$	10	{−14,−12,−10,−8,−6,−4,−2,0,2,4,6,8,10,12,14,16,128}	16

## Data Availability

No new data were created or analyzed in this study. Data sharing is not applicable to this article.
